# Symptomatic late saphenous vein graft failure in coronary artery bypass surgery

**DOI:** 10.1093/icvts/ivad052

**Published:** 2023-04-04

**Authors:** Mikael Janiec, Axel Dimberg, Rickard P F Lindblom

**Affiliations:** Department of Cardiothoracic Surgery and Anesthesia, Uppsala University Hospital, Uppsala, Sweden; Department of Surgical Sciences, Uppsala University, Uppsala, Sweden; Department of Cardiothoracic Surgery, Karolinska University Hospital, Stockholm, Sweden; Department of Cardiothoracic Surgery and Anesthesia, Uppsala University Hospital, Uppsala, Sweden; Department of Surgical Sciences, Uppsala University, Uppsala, Sweden

**Keywords:** Coronary artery bypass grafting, Coronary artery bypass grafting, Graft failure

## Abstract

**OBJECTIVES:**

Coronary artery bypass grafting for advanced coronary artery disease is a well-established procedure with excellent long-term results. The issue of saphenous vein graft (SVG) performance and its relation to clinical symptoms and thereby the potential for improvement by using superior grafts are still not fully understood. We aim to estimate the contribution of late SVG failure to the long-term outcome.

**METHODS:**

A study population operated between 1997 and 2020, with an internal thoracic artery with a single distal anastomosis and 1, 2 or 3 distal SVG anastomoses, was isolated from the Swedish Web System for Enhancement and Development of Evidence-Based Care in Heart Disease Evaluated According to Recommended Therapies registry. Data regarding postoperative clinically driven coronary angiography and status of bypass grafts were collected.

**RESULTS:**

The study population consisted of 44 951 patients. Clinically driven angiography occurred in 10.1% (9.5–10.8), 7.9% (7.6–8.3) and 7.1% (6.7–7.5), respectively, of patients within 3 years and 23.6% (22.6–24.5), 20.0% (19.5–20.6) and 17.5% (16.9–18.2), respectively, of patients within 10 years after surgery. Excluding the first 3 postoperative years, no failed SVGs were found in >75%, 60% and 45%, respectively, of cases when an angiography was performed in the first 10 years after surgery.

**CONCLUSIONS:**

The results suggest that the risk of symptomatic graft failure due to vein graft disease during the first 10 years after surgery is in the range of 1–2% for every grafted coronary vessel and provide an estimate for the upper limit of the improvements in results that could be achieved by replacing SVGs with superior grafts.

**Figure:**
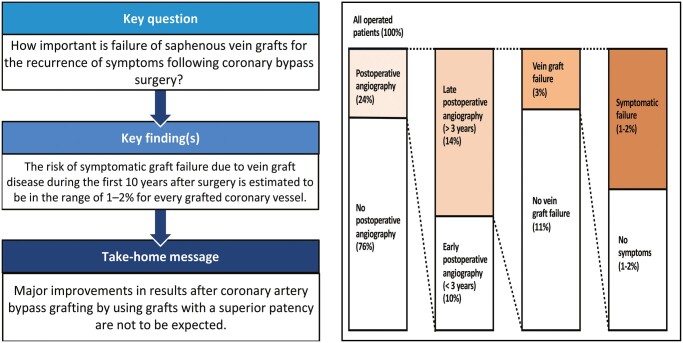

## INTRODUCTION

Saphenous vein grafts (SVGs), the most commonly used graft in coronary artery bypass grafting (CABG), are subject to vein graft disease [1], and their reduced long-term patency as compared to left internal thoracic artery (ITA) grafts is well established [2, 3]. A considerable effort has been made to improve results by replacing SVGs with arterial grafts with superior patency [4–6], but a corresponding improvement in clinical outcome has, however, not consistently been observed [7]. To estimate to which extent an improvement in graft patency also will translate into an improved clinical outcome, the contribution of late SVG graft failure to the recurrence of symptoms must first be quantified. Recurrent symptoms of coronary artery disease after CABG surgery can, in addition to SVG failure, be the result of progression of atherosclerosis in the coronary arteries, including microvascular dysfunction and in-stent stenosis in previously placed stents, or failure of the ITA graft. Significant vein graft stenoses rarely develop before 3 years after grafting [1, 8] and symptomatic SVG failures observed early after surgery are therefore also likely to be unrelated to vein graft disease, and instead caused by technical failures related to the anastomosis or graft kinking, low flow due to competitive flow or thrombosis. An improved graft patency conferred by e.g. arterial conduits may thus not necessarily have a large enough effect size to result in an observable clinical improvement.

We conducted a nationwide observational study of patients operated with CABG in Sweden between 1997 and 2020. We aim to quantify the mortality, incidence of first postoperative angiography and evaluate the reported patency of the SVG and ITA graft to assess the contribution of late SVG failure to the long-term outcome. We believe that a comprehendible model for investigating the association between vein graft disease and long-term clinical outcome can be achieved by specifically investigating CABG with a single and non-sequential SVG as secondary graft to ITA in a large cohort. To confirm the validity of the results and their applicability in the more common setting of multiple SVG vessel grafting, we also study patients with 2 and 3 distal SVG anastomoses.

## PATIENTS AND METHODS

### Ethics statement

The study was approved by the institutional review board, Swedish Ethical Review Authority, Gothenburg, Sweden (Dnr 2020-06252). Informed consent was not required and also not possible to obtain due to the anonymity of the participants.

### Data sources and study population

No informed consent was required. The Swedish personal identity number allows nationwide registries with the possibility of long-term follow-up. The Swedish Web System for Enhancement and Development of Evidence-Based Care in Heart Disease Evaluated According to Recommended Therapies (SWEDEHEART) registry [9] provides baseline data on all patients undergoing cardiac surgery in Sweden as well as records of all angiographies. Registered data include information on the degree of stenosis of individual grafts. Patients with a permanent residence in Sweden between 40 and 80 years of age with no congenital malformations or previous cardiac surgery, who underwent isolated CABG between 1997 and 2020 using a single ITA anastomosis and 1, 2 or 3 distal anastomoses using the saphenous vein as graft, were identified within the SWEDEHEART registry. It was not possible to distinguish if separate or sequential grafts were used in patients with multiple distal SVG anastomoses. We obtained baseline characteristics at the time of surgery and data on postoperative angiographies for included individuals. The only patients who are believed to have been lost to follow-up are individuals undergoing coronary angiography outside of Sweden. Estimated mortality for a cohort matched for sex and year of birth in the general Swedish population was calculated using data provided by the government agency for official statistics (Statistics Sweden).

### Outcomes

The elapsed time from the operation to death and first clinically driven angiography was used as end points. These outcomes represent robust, easily definable, clinically relevant events that are likely to have a high correlation with symptoms related to coronary artery disease and have been used previously to evaluate results after CABG [10, 11]. Date of death was obtained from the national population registry, and dates for CABG procedures and postoperative angiographies were available in SWEDEHEART. The clinical indication for each angiography and the presence of failed graft were registered by the angiographer. From 2005 and onwards, data on the degree of stenosis for individual grafts are also often available. A graft with a reported total occlusion or a stenosis of ≥70% was classified as failed.

### Statistical analysis

Data management and statistical analyses were performed with the use of R version 3.1.3 (R Foundation for Statistical Computing, Vienna, Austria). Patient characteristics were described by using frequencies and percentages for categorical variables and means and standard deviations for continuous variables. Patients were followed from the date of surgery until the date of death from any cause or the end of follow-up (15 September 2021). The Kaplan–Meier method was used to illustrate cumulative survival, and the cumulative incidence function was used to estimate the cumulative incidence of first postoperative angiography using the R package ‘survival’. Hazard functions were estimated for the risk of angiography using the R package ‘muhaz’. Indications for the first angiography and the presence of failed grafts were reported using frequencies and percentages.

## RESULTS

### Study population and baseline characteristics

In total, 71 919 patients with a permanent residence in Sweden between 40 and 80 years of age, with no congenital malformations or previous cardiac surgery, underwent isolated CABG with a single ITA anastomosis between 1997 and 2020. Out of the patients with complete and incontrovertible data on graft type and number of distal anastomoses, 8518 also received 1 distal SVG anastomosis, 21 842 received 2 distal SVG anastomoses and 14 591 received 3 distal SVG anastomoses. Baseline characteristics are shown in Table 1.

**Table 1:** Indications for the first postoperative angiography

	One SVG	Two SVGs	Three SVGs
Total number of patients	2407	5508	3370
Indications, % (*n*)			
STEMI	6.1 (147)	5.7 (314)	4.9 (215)
NSTEMI or unstable angina	42.7 (1027)	42.4 (2337)	43.4 (1462)
Stable angina	41.5 (988)	40.6 (2240)	38.0 (1279)
Other	9.7 (245)	11.2 (617)	12.3 (414)

The indications for the first postoperative angiography for patients with single ITA and 1, 2 or 3 distal SVG anastomoses. The other indications are: valvular disease, arrhythmia, other or unknown.

ITA: internal thoracic artery; NSTEMI: non-ST segment elevation myocardial infarction; SVGs: saphenous vein grafts; STEMI: ST segment elevation myocardial infarction.

### Mortality

#### One distal saphenous vein graft anastomosis

**Figure 1:**
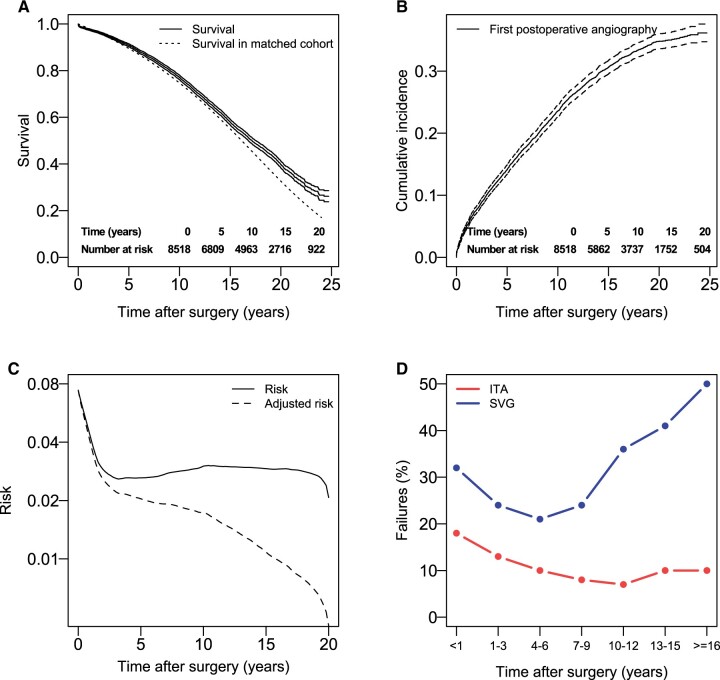
Outcomes for patients operated CABG with 1 distal ITA anastomosis and 1 distal SVG anastomosis. (**A**) Kaplan–Meier estimates and 95% confidence intervals of survival and estimated survival in a cohort matched for age and sex. Tables of number of patients at risk are also shown. (**B**) Cumulative incidence estimates and 95% confidence intervals of first clinically driven angiography. Tables of number of patients at risk are also shown. (**C**) Hazard function for the risk first postoperative angiography. The adjusted hazard function represents the temporal distribution of all first postoperative angiographies for the cohort of operated patients. (**D**) The frequency of failed SVG and ITA grafts found during angiography for different time intervals after surgery. CABG: coronary artery bypass grafting; ITA: internal thoracic artery; SVG: saphenous vein graft.

Thirty-day mortality was 0.95% (*n* = 81), and mortality during follow-up occurred in 42.4% (*n* = 3616) of patients. Survival [95% confidence interval (CI)] at 3, 10 and 20 years was 94.7% (94.1–95.1), 77.1% (76.1–78.0) and 39.8% (38.3–41.2), respectively (Fig. 1A). Expected survival for an age- and sex-matched cohort from the general population 3, 10 and 20 years after surgery was 94.1%, 74.7% and 32.6%, respectively (Fig. 1A).

#### Two distal saphenous vein graft anastomoses

**Figure 2:**
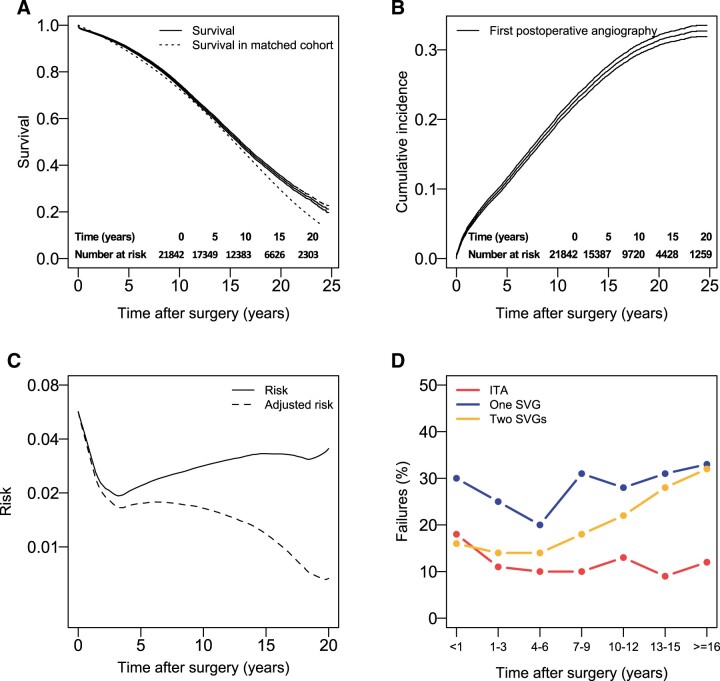
Outcomes for patients operated with CABG with 1 distal ITA anastomosis and 2 distal SVG anastomoses. (**A**) Kaplan–Meier estimates and 95% confidence intervals of survival and estimated survival in a cohort matched for age and sex. Tables of number of patients at risk are also shown. (**B**) Cumulative incidence estimates and 95% confidence intervals of first clinically driven angiography. Tables of number of patients at risk are also shown. (**C**) Hazard function for the risk first postoperative angiography. The adjusted hazard function represents the temporal distribution of all first postoperative angiographies for the cohort of operated patients. (**D**) The frequency of failed SVG and ITA grafts found during angiography for different time intervals after surgery. Failure rates for 1 and 2 grafts estimated by using patency data from the group with 1 SVG is also shown. CABG: coronary artery bypass grafting; ITA: internal thoracic artery; SVG: saphenous vein graft.

Thirty-day mortality was 1.1% (*n* = 235) and mortality during follow-up occurred in 47.3% (*n* = 10 339) of patients. Survival (95% CI) at 3, 10 and 20 years was 94.2% (93.9–94.5), 74.2% (73.5–74.8) and 34.6% (33.7–35.4), respectively (Fig. 2A). Expected survival for an age- and sex-matched cohort from the general population 3, 10 and 20 years after surgery was 93.6%, 72.5% and 29.2%, respectively (Fig. 2A).

#### Three distal saphenous vein graft anastomoses

**Figure 3:**
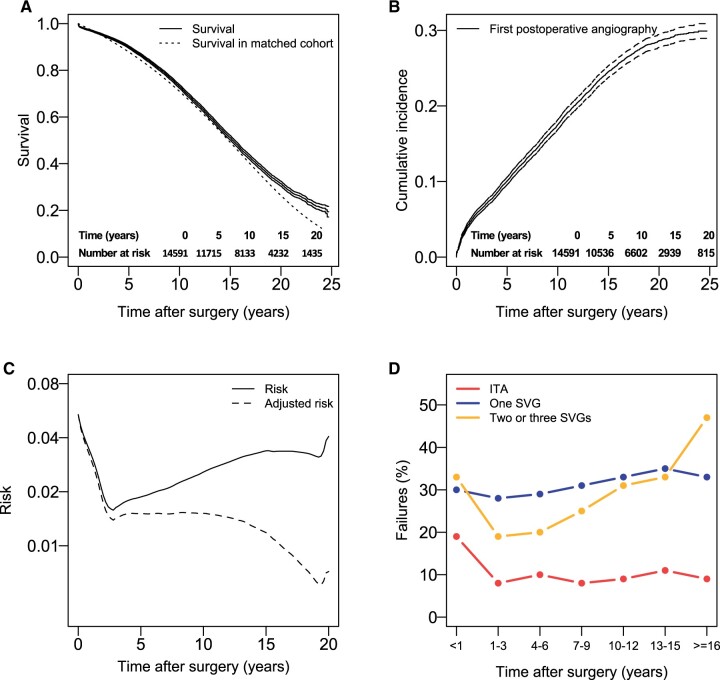
Outcomes for patients operated with CABG with 1 distal ITA anastomosis and 3 distal SVG anastomoses. (**A**) Kaplan–Meier estimates and 95% confidence intervals of survival and estimated survival in a cohort matched for age and sex. Tables of number of patients at risk are also shown. (**B**) Cumulative incidence estimates and 95% confidence intervals of first clinically driven angiography. Tables of number of patients at risk are also shown. (**C**) Hazard function for the risk first postoperative angiography. The adjusted hazard function represents the temporal distribution of all first postoperative angiographies for the cohort of operated patients. (**D**) The frequency of failed SVG and ITA grafts found during angiography for different time intervals after surgery. Failure rates for 1 and 2 grafts estimated by using patency data from the group with 1 SVG is also shown. CABG: coronary artery bypass grafting; ITA: internal thoracic artery; SVG: saphenous vein graft.

Thirty-day mortality was 1.0% (152) and mortality during follow-up occurred in 49.4% (*n* = 7201) of patients. Survival (95% CI) at 3, 10 and 20 years was 94.2% (93.8–94.6), 73.0% (72.2–73.8) and 31.4% (30.4–32.5), respectively (Fig. 3A). Expected survival for an age- and sex**-**matched cohort from the general population 3, 10 and 20 years after surgery was 93.2%, 70.9% and 26.3%, respectively (Fig. 3A).

### Clinically driven angiography

#### One distal saphenous vein graft anastomosis

Postoperative clinically driven angiography occurred in 28.2% (*n* = 2407) of patients. The cumulative incidence (95% CI) at 3, 10 and 20 years for the first clinically driven postoperative angiography was 10.1% (9.5–10.8), 23.6% (22.6–24.5) and 34.8% (33.6–36.0), respectively (Fig. 1B). By adjusting the risk with the surviving population, the distribution of all first postoperative angiographies that will occur in the operated population can be estimated (Figs 1–3C). The indications for the angiography for patients in all 3 groups are presented in Table 1.

#### Two distal saphenous vein graft anastomoses

Postoperative clinically driven angiography occurred in 25.2% (*n* = 5508) of patients. The cumulative incidence (95% CI) at 3, 10 and 20 years for the first clinically driven postoperative angiography was 7.9% (7.6–8.3), 20.0% (19.5–20.6) and 31.4% (30.7–32.1), respectively (Fig. 2B).

#### Three distal saphenous vein graft anastomoses

Postoperative clinically driven angiography occurred in 23.1% (*n* = 3370) of patients. The cumulative incidence (95% CI) at 3, 10 and 20 years for the first clinically driven postoperative angiography was 7.1% (6.7–7.5), 17.5% (16.9–18.2) and 28.6% (27.8–29.5), respectively (Fig. 3B).

### Failed grafts

#### One distal saphenous vein graft anastomosis

From 2005 and onwards, data on individual grafts are registered, and 1401 of 2407 angiographies had data available regarding the degree of stenosis of individual grafts. The 2.6% (*n* = 37) of cases where the number of SVGs was found to be 2 or more, indicating a misclassification at time of surgery, was removed from following analysis. The targets of the ITA and SVGs are presented in Table 2 for all of the groups. A failed ITA graft was reported in 11% (*n* = 138) and a failed SVG in 31% (403) of the angiographies with patency data (Supplementary Material, Table SB). The probability of finding a failed ITA graft was similar in all of the groups (Supplementary Material, Table SB–SD). The probability of finding any failed grafts was higher in the first year after surgery. For SVGs, the failure rate increased with time after being relatively stable for the first 10 postoperative years (Fig. 1D).

**Table 2:** Targets of grafts reported during first postoperative angiography

		One SVG	Two SVGs	Three SVGs
Total number of patients		1364	3312	2056
Grafts	Distal anastomosis, % (*n*)			
ITA	LAD	90.1 (1239)	90.6 (3001)	86.8 (1785)
	Diagonal	2.7 (37)	3.0 (99)	2.2 (45)
	Other	1.5 (21)	0.8 (28)	1.8 (37)
	Unknown	4.9 (67)	5.6 (184)	9.2 (189)
SVG(s)	Marginal	42.8 (584)	36.4 (2414)	30.3 (1866)
	Intermediary	6.0 (82)	5.2 (347)	4.7 (290)
	PDA	20.3 (277)	28.9 (1917)	23.6 (1458)
	RCA	6.7 (92)	5.9 (388)	3.7 (277)
	Diagonal	15.2 (208)	14.6 (966)	20.6 (1268)
	Other	6.2 (85)	3.5 (230)	6.5 (404)
	Unknown	2.6 (36)	5.5 (362)	10.6 (655)

Targets of grafts reported during first postoperative angiography for patients with single ITA and 1, 2 or 3 distal SVG anastomoses.

ITA: internal thoracic artery; LAD: left anterior descending artery; PDA: posterior descending artery; RCA: right coronary artery; SVG: saphenous vein graft.

#### Two distal saphenous vein graft anastomoses

Of 5508 angiographies, 3384 had data available regarding the degree of stenosis of individual grafts. 2.1% (*n* = 72) of cases where the number of SVGs was found to be 3 or more were removed from the following analysis. A failed SVG was reported in 28% (*n* = 889) and 2 failed SVGs in 20% (627) of the angiographies (Supplementary Material, Table SC).

#### Three distal saphenous vein graft anastomoses

Of 3370 angiographies, 2061 had data available regarding the degree of stenosis of individual grafts. 0.2% (*n* = 5) of cases where the number of SVGs was found to be 4 or more were removed from the following analysis. A failed SVG was reported in 31% (632), 2 in 16% (331) and 3 in 12% (249) of angiographies (Supplementary Material, Table SD).

## DISCUSSION

We present outcome data from a large cohort on CABG-operated patients on mortality and clinically driven angiography, including reported individual graft patency. We believe that we have a comprehensible model for investigating the association between vein graft disease and clinical outcome and that we thereby can estimate the contribution from late SVG failures to long-term outcome.

Failure rates of ITA grafts in early protocol angiography have been reported to be 5–8% [3, 12, 13] and 15% 10 years after surgery [3]. In clinically driven angiography, it has been found to be as high 27% within the first year after surgery [14]. In our study, we found for all groups that 18–19% of ITA grafts had failed in clinically driven catheterizations during the first postoperative year and that 10–11% had failed later after surgery. These results suggest that early failures due to technical errors or thrombosis often are symptomatic and are discovered within the first years after surgery and that it is more uncommon that ITA grafts develop symptomatic failures with time.

Most SVG failures occur without symptoms and do not seem to influence mortality and cardiovascular events after CABG surgery [15, 16]. During protocol angiography early after CABG, the reported failure rates have ranged between 12% and 43% [12, 13, 15, 17–19]. The main determinants of SVG failure during the first postoperative year appear to be a small vessel diameter, reduced wall motion of the vessel-dependent myocardial region and the right coronary artery as target vessel [3, 16, 18], in particular when the vessel has a chronic total occlusion [19]. This indicates that it is grafts supplying less important myocardial regions or regions supplied with collateral arteries that fail early and perhaps also why they generally do so without symptoms or prognostic consequences. A haemodynamically important stenosis rarely develops as the result of vein graft disease in the first 3 years after grafting [1]. The graft attrition rate has been reported to be 1–2% per year between 1 and 6 years after surgery, and 4% per year between 6 and 10 years after surgery [8, 20, 21]. Symptomatic graft failures have thus a more infrequent occurrence and the incidence and prognostic factors have been more difficult to study. As asymptomatic SVG failures are very common and will be discovered in conjunction with a later angiography regardless of indication, it may not be possible do determine what impact a failed graft has on the current symptom and the importance of a discovered failed graft could be overestimated. In the context of CABG with multiple grafts, it will be even more difficult to assess the importance of one or more graft failures.

In our study, a failed SVG was found in 31% of all catheterizations in the group with 1 SVG. Between 4 and 7 years after surgery, the failure rate was at its lowest with 21%, giving an estimate of the upper limit of the incidence of asymptomatic early failures. As expected the probability of finding a failed SVG increased with time and reached 50% for catheterizations performed >16 years postoperatively. The probability of finding a failed graft for different time intervals after surgery in our study (Figs 1–3D) is consistent with a high incidence of early asymptomatic SVG failures and symptomatic technical failures, followed by an increasing risk of SVG failure, starting 3–5 years after surgery [1, 8, 15].

The probability of finding multiple failed grafts was higher than the expected distribution of graft failures under the assumption that every SVG fails independently. If one failed graft is found, there was a relatively high probability to find that the second and third grafts had failed as well. This could imply that patients with multiple grafts develop symptoms more often or that patients with a single failure have a lower risk of developing symptoms when multiple vessels are grafted. It may also indicate that the SVGs do not fail independently, e.g. by the use of sequential grafts, factors related to the surgical technique or patient-related factors such as graft quality or tendency to develop vein graft disease.

For a large part of about 30–35% of patients who will experience an angiography postoperatively, the first angiography will occur already within the first 3 years after surgery (Figs 1–3B), when significant vein graft disease has not yet had time to develop [1], and most of symptomatic graft failure can be attributed to thrombosis or technical failures. For the remaining patients, the angiography seems unrelated to SVG failure during the first 10 years postoperatively in about 75% of cases in the group with a single SVG, 60% in the group with two and 45–50% in the group with three distal SVG anastomoses (Supplementary Material, Tables SB–SD). In a significant number of patients where a failed SVG is present, the discovered failure is, as previously discussed, asymptomatic and unrelated to the symptoms that preceded the angiography. Using 12% [13] as a low approximation and 21% (Supplementary Material, Table SB) as a high approximation for asymptomatic early SVG failures, a probable range of symptomatic failures can also be estimated. If about 30% of SVGs found during clinically driven angiography have failed, between 1/3 and 2/3 of these will therefore probably be unrelated to the symptoms.

**Figure 4:**
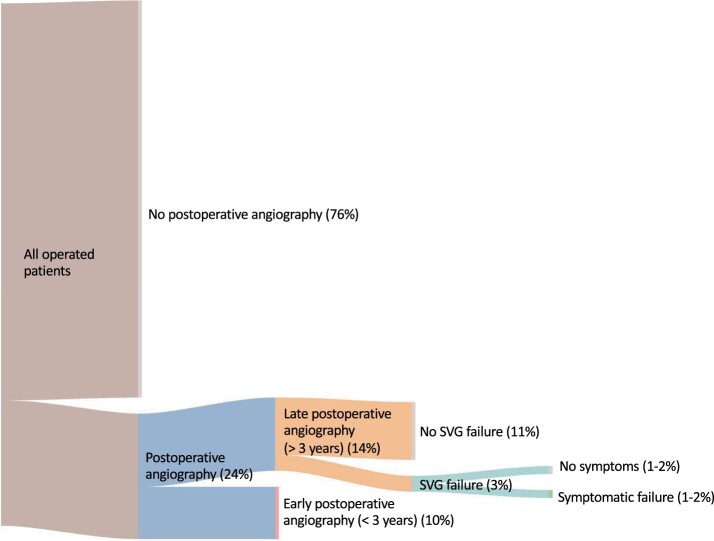
Figure illustrating the estimated distribution of different outcomes 10 years after surgery for patients operated with CABG with 1 distal ITA anastomosis and 1 distal SVG anastomosis. The proportion of patients that will experience a late (>3 years postoperatively) symptomatic SVG failure is estimated to be in the rage of 1–2% of the operated population. CABG: coronary artery bypass grafting; ITA: internal thoracic artery; SVG: saphenous vein graft.

For the patients with 1 SVG, a failed graft is found in 3% of the operated population in conjunction with a postoperative angiography between 3 and 10 years after surgery. If we estimate that between 1/3 and 2/3 of all the failures found are symptomatic, only 1–2% will experience a symptomatic late SVG failure that to some extent could be influenced by using grafts with a superior patency (Fig. 4). With the increasing complexity of additional SVGs present, it is increasingly difficult to estimate the risk of late symptomatic SVG failure. For the patients with 2 and 3 SVG anastomoses, at least 1 failed graft is found in 5% and 6% of the operated population, respectively, in conjunction with a postoperative angiography between 3 and 10 years after surgery. Assuming again that between 1/3 and 2/3 of a discovered single failure is related to the symptoms preceding the angiography, and that all multiple failures are symptomatic, this will constitute <4% and <5%, respectively, of the operated population (Supplementary Material, Figs SA and SB). The risk of symptomatic graft failure due to vein graft disease during the first 10 years after surgery should therefore not be higher than 1–2% for every vessel grafted with SVGs.

As most catheterizations will occur within the first 10 postoperative years (Figs 1–3B), a large part of the total number of postoperative angiographies will always be unrelated to late SVG failure, even though there will be a very high probability of finding failed grafts late after surgery (Figs 1–3D).

The results provide important insights into why it has been difficult to demonstrate a superior clinical outcome during the first 10 years after surgery for patients where an SVG was replaced with an arterial graft [7]. Even if a superior graft would prevent all symptomatic failures, the improvements in outcome during the first 10 years may be of a magnitude beneath the level of detection, or partially be eclipsed by the competing risks of patients reaching study end points due to events with causes unrelated to SVG failure. Furthermore, there is a risk that the achievable advantages may be lost even with a very small increase in the risk of early technical failures as a consequence of a more technically demanding procedure. The results also highlight the significance of atherosclerotic progression in the coronary arteries and the importance of optimal secondary prevention.

### Limitations

The study is observational and the design does not allow for any causal inference to be established. The results are limited by the availability, quality and completeness of the registry data. The long inclusion period could significantly impact the generalizability. The target vessel and whether the left or right ITA was used is not registered at base-line. The degree of stenosis in the bypassed vessels at the time of surgery is also unknown. Some of the early postoperative angiographies may have been part of a planned hybrid procedure of multivessel disease or due to an inability to find or to graft a target vessel during surgery. The classification of the degree of stenosis of grafts was completely operator dependent. It is not possible to know the importance of graft failure in patients who died suddenly from an ischaemic event without an angiography.

## Supplementary Material

ivad052_Supplementary_Data

## Data Availability

The analytic methods will be made available from the corresponding author to other researchers for purposes of reproducing the results or replicating the procedure. The data will be made available with the permission of SWEDEHEART. **Mikael Janiec:** Conceptualization; Data curation; Formal analysis; Investigation; Methodology; Software; Validation; Visualization; Writing—original draft; Writing—review & editing. **Axel Dimberg:** Conceptualization; Methodology; Visualization; Writing—original draft; Writing—review & editing. **Rickard P.F. Lindblom:** Conceptualization; Project administration; Supervision; Writing—original draft; Writing—review & editing. Interdisciplinary CardioVascular and Thoracic Surgery thanks Cem H. Alhan, Luca Di Marco, Yoshito Inoue and the other anonymous reviewer(s) for their contribution to the peer review process of this article.

## ABBREVIATIONS

CABG: Coronary artery bypass grafting
ITA: Internal thoracic artery
SVG: Saphenous vein graft
SWEDEHEART: Swedish Web System for Enhancement and Development of Evidence-Based Care in Heart Disease Evaluated According to Recommended Therapies
