# Supplementary material for: Symptomatic late saphenous vein graft failure in coronary artery bypass surgery
Source: Interdiscip Cardiovasc Thorac Surg. 2023 Apr 4;36(4):ivad052. doi: 10.1093/icvts/ivad052 (PMC10081881; doi:10.1093/icvts/ivad052)
Supplement: ivad052_Supplementary_Data [file ivad052_supplementary_data.zip › Supplement figure legends.docx]

**Supplement figure legends**

**Supplement figure A.** Figure illustrating the estimated distribution of different outcomes 10 years after surgery for patients operated with CABG with one distal ITA anastomosis and two distal SVG anastomosis. The proportion of patients that will experience a late (>3 years postoperatively) symptomatic SVG failure is estimated to be in the rage of 3–4% of the operated population. CABG coronary artery bypass grafting; ITA internal thoracic artery; SVG saphenous vein graft.

**Supplement figure B.** Figure illustrating the estimated distribution of different outcomes 10 years after surgery for patients operated with CABG with one distal ITA anastomosis and three distal SVG anastomosis. The proportion of patients that will experience a late (>3 years postoperatively) symptomatic SVG failure is estimated to be in the rage of 4–5% of the operated population. CABG coronary artery bypass grafting; ITA internal thoracic artery; SVG saphenous vein graft.
