# Supplementary material for: Symptomatic late saphenous vein graft failure in coronary artery bypass surgery
Source: Interdiscip Cardiovasc Thorac Surg. 2023 Apr 4;36(4):ivad052. doi: 10.1093/icvts/ivad052 (PMC10081881; doi:10.1093/icvts/ivad052)
Supplement: ivad052_Supplementary_Data [file ivad052_supplementary_data.zip › Supplement table A.docx]

**Supplement table A**

|  | One SVG | Two SVGs | Three SVGs |
| --- | --- | --- | --- |
| Total study population | 8518 | 21842 | 14591 |
| Mean follow-up (years) | 14.91 (6.64) | 15.22 (6.69) | 15.40 (6.66) |
| Mean (SD) age (years) | 65.77 (8.53) | 66.65 (8.37) | 67.01 (8.25) |
| Female | 2013 (23.6%) | 4746 (21.7%) | 2570 (17.6%) |
| Mean (SD) body mass index (mg/kg2) | 27.48 (6.61) | 27.52 (5.73) | 27.42 (4.91) |
| Body mass index not recorded | 773 (9.1%) | 2386 (10.9%) | 1918 (13.1%) |
| Diabetes | 1768 (21.6%) | 5183 (25.0%) | 3481 (24.9%) |
| Presence of diabetes not recorded | 351 (4.1%) | 1091 (5.0%) | 615 (4.2%) |
| Renal impairment: |  |  |  |
| Normal, (CC >85 ml/min) | 3478 (46.0%) | 8262 (43.7%) | 5271 (43.0%) |
| Moderately impaired (50-85 ml/min) | 3468 (45.9%) | 8986 (47.5%) | 5891 (48.0%) |
| Severely impaired (<50 ml/min) off dialysis | 554 (7.3%) | 1547 (8.2%) | 1045 (8.5%) |
| On dialysis | 55 (0.73%) | 111 (0.59%) | 57 (0.46%) |
| Renal impairment not recorded | 963 (11.3%) | 2936 (13.4%) | 2327 (15.9%) |
| COPD | 376 (6.7%) | 986 (7.2%) | 569 (6.5%) |
| COPD not recorded | 2916 (34.2%) | 8170 (37.4%) | 5887 (40.3%) |
| Extracardiac arteriopathy | 402 (7.1%) | 1118 (8.1%) | 705 (8.1%) |
| Extracardiac arteriopathy not recorded | 2893 (34.0%) | 8066 (36.9%) | 5842 (40.0%) |
| Neuromuscular disability | 264 (5.0%) | 758 (5.8%) | 464 (5.4%) |
| Neuromuscular disability not recorded | 3239 (38.0%) | 8793 (40.3%) | 5986 (41.0%) |
| Ejection fraction: |  |  |  |
| Normal | 1659 (72.4%) | 4057 (70.2%) | 2746 (69.2%) |
| 30%-50% | 522 (22.8%) | 1429 (24.7%) | 1047 (26.4%) |
| <30% | 112 (4.9%) | 293 (5.1%) | 177 (4.5%) |
| Ejection fraction not recorded | 6225 (73.1%) | 16063 (73.5%) | 10621(72.8%) |
| MI last 90 days | 2050 (36.4%) | 5647 (41.0%) | 3839 (43.7%) |
| Prior MI not recorded | 2888 (33.9%) | 8058 (36.9%) | 5815 (39.9%) |
| Previous PCI | 1396 (25.8%) | 2754 (19.9%) | 1876 (19.5%) |
| Previous PCI not recorded | 3100 (36.4%) | 7987 (36.6%) | 4978 (34.1%) |
| Euroscore: |  |  |  |
| 0-2 | 2604 (39.6%) | 5640 (34.7%) | 3584 (33.8%) |
| 3-5 | 2768 (42.1%) | 7254 (44.6%) | 4788 (45.1%) |
| >5 | 1209 (18.4%) | 3360 (20.7%) | 2238 (21.1%) |
| Euroscore not recorded | 1937 (22.7%) | 5588 (25.6%) | 3981 (27.3%) |
| 30 day mortality | 81 (0.95%) | 235 (1.1%) | 152 (1.0%) |

Baseline characteristics of included patients. SD standard deviation; CC creatinine clearence; COPD chronic obstructive pulmonary disease; PCI percutaneous coronary intervention; MI myocardial infarction.
