# Supplementary material for: Symptomatic late saphenous vein graft failure in coronary artery bypass surgery
Source: Interdiscip Cardiovasc Thorac Surg. 2023 Apr 4;36(4):ivad052. doi: 10.1093/icvts/ivad052 (PMC10081881; doi:10.1093/icvts/ivad052)
Supplement: ivad052_Supplementary_Data [file ivad052_supplementary_data.zip › Supplement table B.docx]

**Supplement table B**

| **Time after surgery (years)** | **Number of patients** | **ITA grafts** |  | **SVGs** |  |
| --- | --- | --- | --- | --- | --- |
|  |  | **Failed** | **No data** | **Failed** | **No data** |
| **<1** | 175 | 18% (31) | 4% (7) | 32% (53) | 6% (11) |
| **1-3** | 256 | 13% (31) | 7% (18) | 24% (60) | 3% (7) |
| **4-6** | 220 | 10% (21) | 4% (9) | 21% (46) | 1% (3) |
| **7-9** | 236 | 8% (17) | 6% (14) | 24% (55) | 3% (8) |
| **10-12** | 222 | 7% (14) | 3% (7) | 36% (78) | 3% (6) |
| **13-15** | 145 | 10% (14) | 4% (6) | 41% (57) | 4% (6) |
| **≥16** | 110 | 10% (10) | 12% (13) | 50% (54) | 3% (3) |
|  |  |  |  |  |  |
| **All** | 1364 | 11% (138) | 5% (74) | 31% (403) | 3% (44) |

Patients operated with one distal ITA anastomosis and one distal SVG anastomosis. Frequency of reported failed grafts at the first post-operative clinically-driven angiography performed at different time-intervals after surgery. ITA internal thoracic artery; SVG saphenous vein graft.
