# Supplementary material for: Symptomatic late saphenous vein graft failure in coronary artery bypass surgery
Source: Interdiscip Cardiovasc Thorac Surg. 2023 Apr 4;36(4):ivad052. doi: 10.1093/icvts/ivad052 (PMC10081881; doi:10.1093/icvts/ivad052)
Supplement: ivad052_Supplementary_Data [file ivad052_supplementary_data.zip › Supplement table C.docx]

**Supplement table C**

| **Time after surgery (years)** | **Number of patients** | **ITA grafts** |  | **SVG grafts** |  |  |
| --- | --- | --- | --- | --- | --- | --- |
|  |  | **Failed** | **No data** | **One failed** | **Two failed** | **No data** |
| **<1** | 355 | 18% (58) | 8% (27) | 30% (102) | 16% (55) | 5% (19) |
| **1-3** | 486 | 11% (48) | 7% (33) | 25% (116) | 14% (63) | 5% (26) |
| **4-6** | 560 | 10% (56) | 5% (26) | 20% (108) | 14% (74) | 6% (33) |
| **7-9** | 636 | 10% (57) | 9% (55) | 31% (192) | 18% (112) | 4% (24) |
| **10-12** | 522 | 13% (62) | 5% (26) | 28% (141) | 22% (110) | 4% (22) |
| **13-15** | 433 | 9% (36) | 6% (28) | 31% (130) | 28% (115) | 4% (19) |
| **≥16** | 320 | 12% (35) | 8% (24) | 33% (98) | 32% (96) | 6% (20) |
|  |  |  |  |  |  |  |
| **All** | 3312 | 11% (352) | 6.6% (219) | 28% (889) | 20% (627) | 5% (163) |

Patients operated with one distal ITA anastomosis and two distal SVG anastomoses. Frequency of reported failed grafts at the first post-operative clinically-driven angiography performed at different time-intervals after surgery. For 8% (281) of patients one of the grafts had incomplete patency data. These were registered as one failed graft or no failed grafts according to the status of the known graft. ITA internal thoracic artery; SVG saphenous vein graft.
