# Supplementary material for: Symptomatic late saphenous vein graft failure in coronary artery bypass surgery
Source: Interdiscip Cardiovasc Thorac Surg. 2023 Apr 4;36(4):ivad052. doi: 10.1093/icvts/ivad052 (PMC10081881; doi:10.1093/icvts/ivad052)
Supplement: ivad052_Supplementary_Data [file ivad052_supplementary_data.zip › Supplement table D.docx]

**Supplement table D**

| **Time after surgery (years)** | **Number of patients** | **ITA grafts** |  | **SVG grafts** |  |  |  |
| --- | --- | --- | --- | --- | --- | --- | --- |
|  |  | **Failed** | **No data** | **One failed** | **Two failed** | **Three failed** | **No data** |
| **<1** | 234 | 19% (39) | 12% (29) | 30% (68) | 18% (40) | 16% (36) | 3% (6) |
| **1-3** | 292 | 8% (20) | 11% (31) | 28% (81) | 11% (31) | 9% (23) | 2% (7) |
| **4-6** | 320 | 10% (28) | 9% (29) | 29% (91) | 13% (40) | 7% (23) | 3% (8) |
| **7-9** | 350 | 8% (25) | 10% (34) | 31% (108) | 13% (45) | 12% (40) | 2% (6) |
| **10-12** | 359 | 9% (29) | 13% (48) | 33% (116) | 17% (59) | 14% (50) | 1% (4) |
| **13-15** | 288 | 11% (27) | 11% (31) | 35% (98) | 19% (53) | 15% (42) | 1% (4) |
| **≥16** | 213 | 9% (17) | 9% (22) | 33% (70) | 30% (63) | 17% (35) | 1% (3) |
|  |  |  |  |  |  |  |  |
| **All** | 2056 | 10% (185) | 11% (224) | 31% (632) | 16% (331) | 12% (249) | 2% (38) |

Patients operated with one distal ITA anastomosis and three distal SVG anastomoses. Frequency of reported failed grafts at the first post-operative clinically-driven angiography performed at different time-intervals after surgery. For 22% (462) of patients one or two of the SVGs had incomplete patency data. These were registered as one, two or no failed grafts according to the status of the known graft(s). ITA internal thoracic artery; SVG saphenous vein graft.
