# Supplementary figures and images for: Symptomatic late saphenous vein graft failure in coronary artery bypass surgery
Source: Interdiscip Cardiovasc Thorac Surg. 2023 Apr 4;36(4):ivad052. doi: 10.1093/icvts/ivad052 (PMC10081881; doi:10.1093/icvts/ivad052)

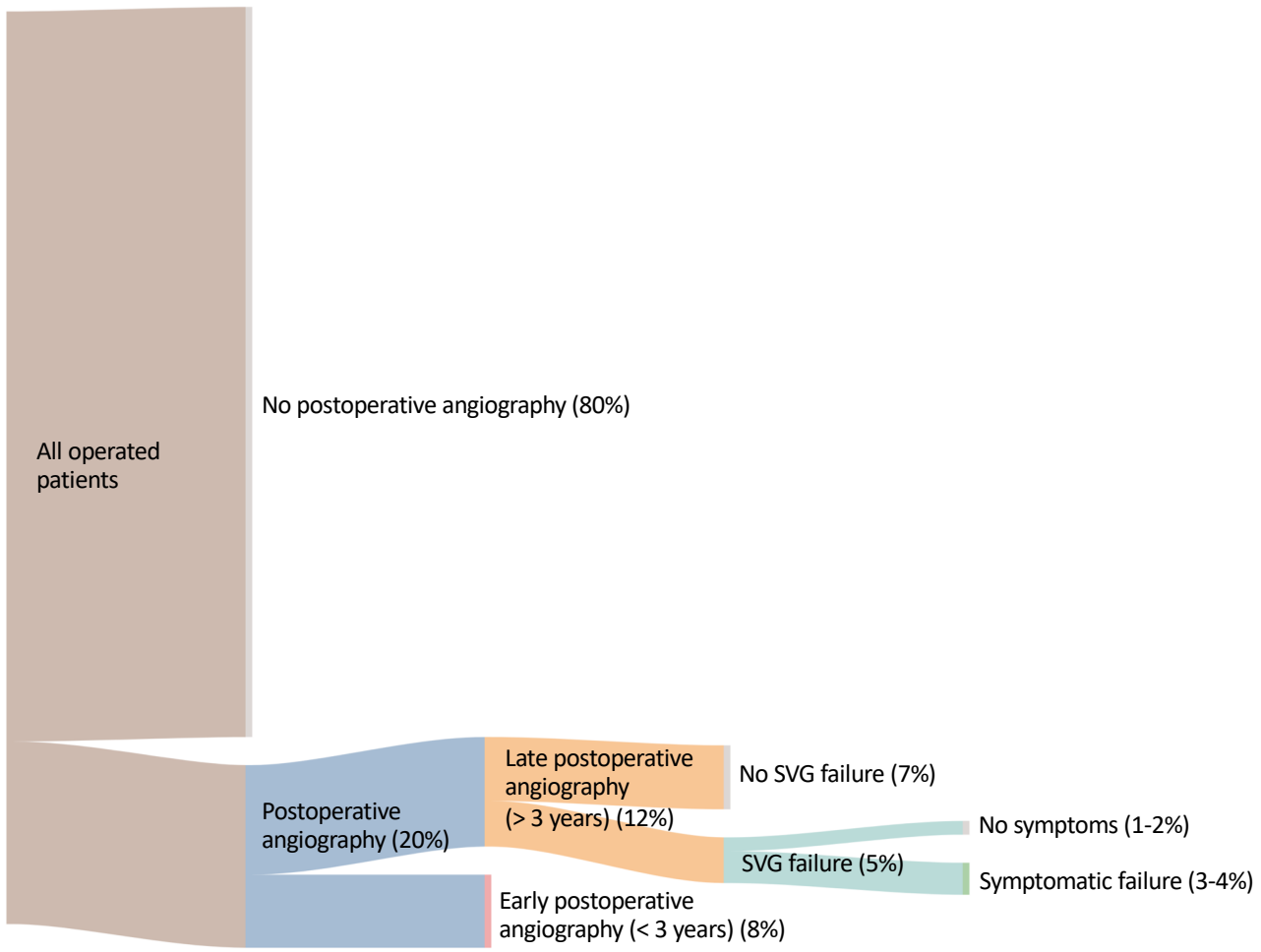

Supplement: ivad052_Supplementary_Data [file ivad052_supplementary_data.zip › Supplement A.pdf]

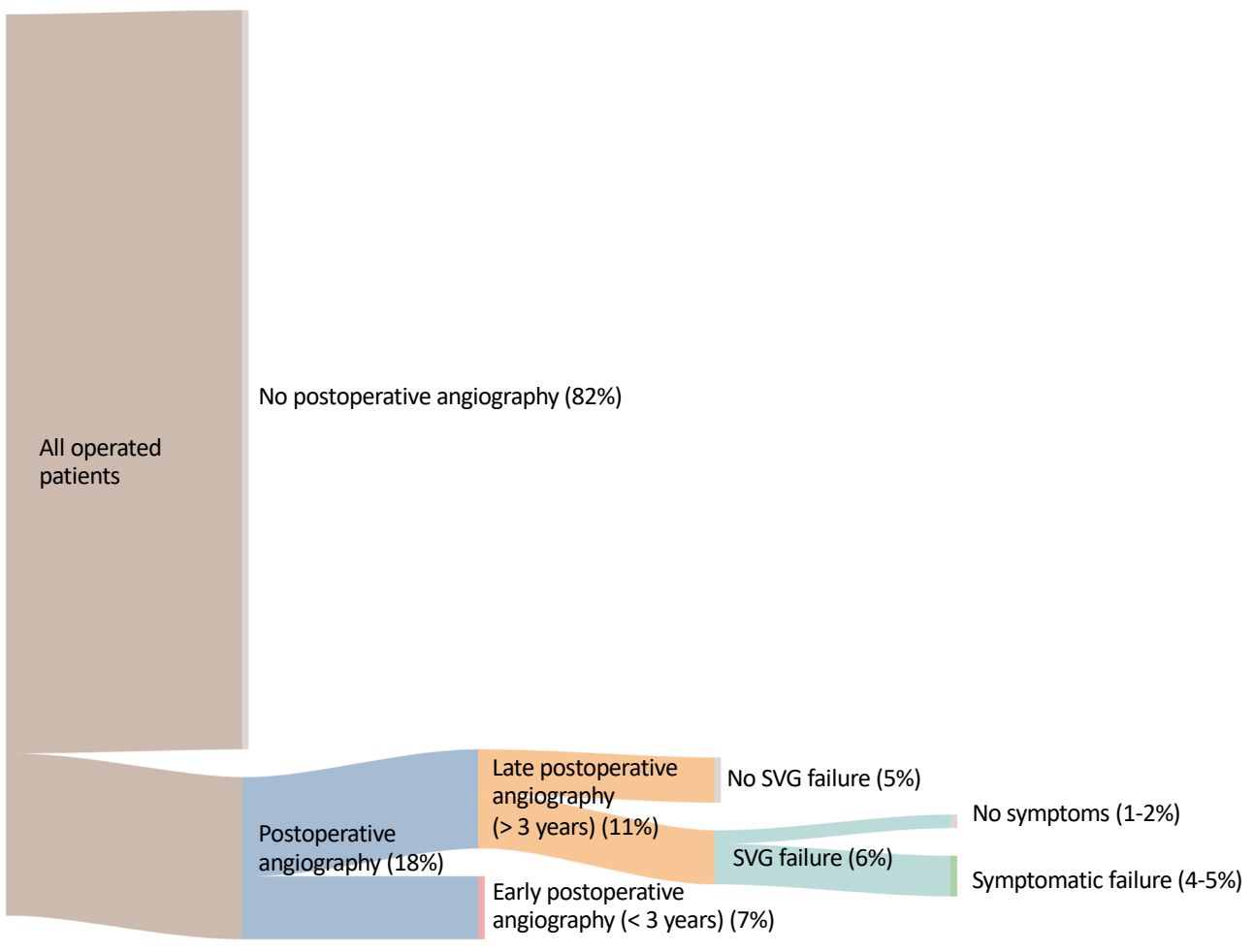

Supplement: ivad052_Supplementary_Data [file ivad052_supplementary_data.zip › Supplement B.pdf]
